# Fabrication of Chalcogenide Glass Based Hexagonal Gapless Microlens Arrays via Combining Femtosecond Laser Assist Chemical Etching and Precision Glass Molding Processes

**DOI:** 10.3390/ma13163490

**Published:** 2020-08-07

**Authors:** Fan Zhang, Qing Yang, Hao Bian, Minjing Li, Xun Hou, Feng Chen

**Affiliations:** 1 State Key Laboratory for Manufacturing System Engineering and Shaanxi Key Laboratory of Photonics Technology for Information, School of Electronic Science and Engineering, Xi’an Jiaotong University, Xi’an 710049, China; zf19900625@stu.xjtu.edu.cn (F.Z.); houxun@mail.xjtu.edu.cn (X.H.); 2 School of Mechanical Engineering, Xi’an Jiaotong University, Xi’an 710049, China; yangqing@mail.xjtu.edu.cn (Q.Y.); liminjing92@stu.xjtu.edu.cn (M.L.); 3 Wuhan National Laboratory for Optoelectronics, Huazhong University of Science and Technology, Wuhan 430074, China

**Keywords:** chalcogenide glass, infrared microlens, femtosecond laser, precision glass molding

## Abstract

Chalcogenide glasses (ChGs) are emerging as critical infrared (IR)-enabled materials in advanced IR optical systems by the wealth of their transparency in the key wide infrared (IR) transmission window. However, fabrication of ChG-based integrated micro-optical components in an efficient and economical way remains a huge challenge. In this paper, a 3D close-packed hexagonal microlens array (MLA) possessing over 6000 convex hexagonal micro-lenslets with the size of tens of micrometers within a footprint of 10 mm × 10 mm on a Ge_20_Sb_15_Se_65_ ChG surface was successfully fabricated via a precise thermal-mechanical molding process. The master mold of ChG MLA was firstly fabricated by a femtosecond laser-assisted chemical etching process and then transferred on to the surface of the ChG via a precision thermo-mechanical molding process, which resulted in a convex MLA. The morphology, imaging and focusing performances of the as-prepared ChG MLA were investigated and demonstrated the advancement of the method. Meanwhile, the IR transmittance and x-ray diffraction image of the ChG MLAs were measured to verify the structural and compositional stability of the ChG under the given molding conditions. The combined results proved a new route to mass production of miniaturized gapless ChG MLAs for advanced infrared micro-optics.

## 1. Introduction

Infrared (IR) optical materials and devices have been gaining increasing attention for their wide application in defense, security and healthcare [1,2,3,4,5]. However, the major barriers to the development of IR optics include having suitable material with low cost and competitive optical properties as well as a suitable corresponding fabrication method for precise and mass production to meet the demand of integrated, miniaturized, high-performance IR devices and systems. The chalcogenide glasses (ChGs) are types of artificial materials made of mainly chalcogen elements (S, Se and Te) bonded with a covalent bond to some other heavy metals and metalloids [6,7,8]. Recently, ChG-based optical components and devices have attracted numerous researchers because of the low cost and unique properties, which include wide transmittance windows (making them suitable for both mid-infrared and long-infrared applications), high refractive indices (*n* = 2.0–3.5) and strong nonlinear properties in the mid-infrared region [9,10,11,12,13,14]. On the contrary, germanium is rare and expensive, silicon has a nonlinear refractive index 100 or even 1000 times lower than that of a chalcogenide glass, and oxide glasses can only be used in the near- and mid-infrared wave band because of the strong infrared absorption of metal-oxygen bond vibrations [15,16,17,18]. Consequently, ChG-based optics are promising in many cutting-edge IR applications, such as near- to long-infrared imaging [19], biologic sensing [20], infrared photodetection [21,22] and infrared optical microcavity for chemical sensors [23].

Precision glass molding is being considered as one of the most promising technologies compared to conventional methods [24] like single point diamond turning and polishing [25,26], chemical mechanical micro-lapping [27], focused ion beam machining and laser processing [28,29], for the mass manufacturing of high-quality, large-area, complex IR components. Considering the low transition temperature (T_g_) and steady physical and chemical properties, ChG is considered an ideal material for precision glass molding technology [30,31]. ChG can be precisely molded into the final shape with high accuracy in one operation [32,33] by offering heat and pressure without large changes to the internal structures. For mass production, precision glass molding is preferred to traditional methods because the molding time is much shorter than the process of single-point diamond turning and polishing [34,35]. Not limited by the size, it can process all kinds of microstructures. In addition, production costs are also decreased because the molds can be reused thousands of times without major wear or deformation. Nevertheless, use of a high-quality master mold with needed features and functions of target IR components for ChG is critical in the precision glass molding process.

Herein, we report the fabrication of microlens arrays (MLAs) on the surface of ChG (Ge_20_Sb_15_Se_65_. NBU-IR1. Ningbo Univ., China) by combining femtosecond laser-assisted chemical etching (FLACE) [36,37] and precision glass molding processes. The FLACE method, which has been demonstrated to be an effective route for high-quality MLAs, is employed to create the concave MLAs on hard and high-temperature-resistant BK7 glass, which is used as a mold in the precision glass molding process. The physical properties of chalcogenide glasses are quite different from the optical glasses commonly used in precision glass molding. Therefore, numerous experiments are needed to determine the optimum parameters for molding ChG. Among all kinds of molding parameters, temperature is the most important parameter as it affects the quality of the molded products [38,39,40]. The surface mass of the microstructure was observed, and the deformation ratios were calculated through several experiments. Precision glass molding was divided into four stages: a heating stage (20 to 340 °C), a pressing stage (340 °C), a slow cooling stage (340 to 200 °C) and a rapid cooling stage (200 to 20 °C). Every stage had precise rates of heating and cooling. The final fabricated IR device was close-packed by 6000 convex micro-lenslets within an area of 10 mm × 10 mm, which had the advantages of high integration, light weight, economical manufacturing and perfect IR imagery. The working band covered a wide region from 2.5 to 22 μm. In addition, by analyzing the IR transmittance and x-ray diffraction (XRD) analysis of ChG MLAs, the structure and composition stability of the glass material under the given molding conditions were verified.

## 2. Materials and Methods

The ChG material used in the experiment was NBU-IR1 (Ge_20_Sb_15_Se_65_. NingboUniv., Ningbo, China). The physical properties of ChG are shown in Table 1 [41].

**Figure 1:**
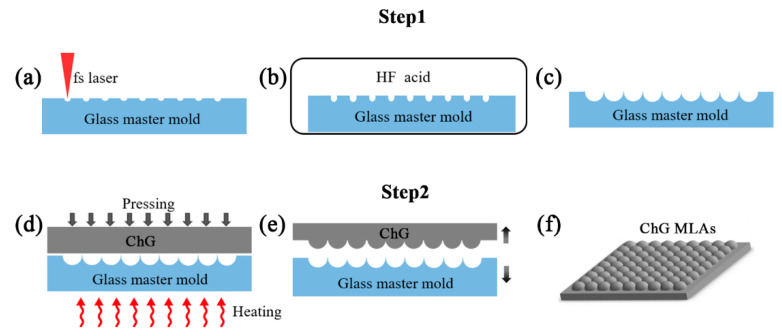
Schematic of the fabrication process. (**a**) An array of laser-exposed craters is produced on BK7 glass by laser irradiation; (**b**) The craters are treated by ultrasonic-assisted HF etching; (**c**) Concave microlens arrays (MLAs) with smooth surfaces are formed; (**d**) The precision glass molding process; (**e**) Separate the chalcogenide glass (ChG) from the mold; (**f**) Convex ChG MLAs.

The fabrication process is shown in Figure 1. The process consists of two steps: the fabrication of the master mold via FLACE, and the glass molding process. The mold can be used thousands of times over without mold repair, so the fabrication of ChG MLA starts from a hard and high-temperature-resistant concave mold, which is made by a highly efficient FLACE technique on a BK7 optical glass substrate. Femtosecond laser pulses (Libra-usp-he, Coherent) [42,43,44], with a central wavelength of 800 nm, pulse duration of 50 fs, repetition rate of 1 kHz and laser power of 5 mW, were used and focused by an objective lens, which had a numerical aperture of 0.5. The high peak intensity of moderately and tightly focused femtosecond laser pulses made this possible through nonlinear multiphoton, avalanche and Coulomb explosions producing permanent structural modifications within a couple of picoseconds. At the same time, the strong shockwave compressed the crystal lattices of the surrounding materials, producing the Lewis base, which could greatly enhance chemical activity in reactions with acids [45]. At the same time, some nanostructures [46] were formed around the craters (Figure 1a). Then, the irradiated sample was put into a solution of 8% (v/v) hydrofluoric (HF) acid at room temperature (Figure 1b). The Lewis base and nanostructures further sped up the chemical reactions by providing more opportunities for the etchant to access the modified region. In the chemical etching process, an ultrasonic bath was necessary to ensure consistency and high speed by removing the modified areas on a liquid–solid interface. The modified regions were etched out at first priority. Then, the micro-craters were expanded and polished progressively, and finally a hard concave MLA mold with a nanometer scale surface roughness (8.5nm) was formed, as shown in Figure 1c.

**Figure 2:**
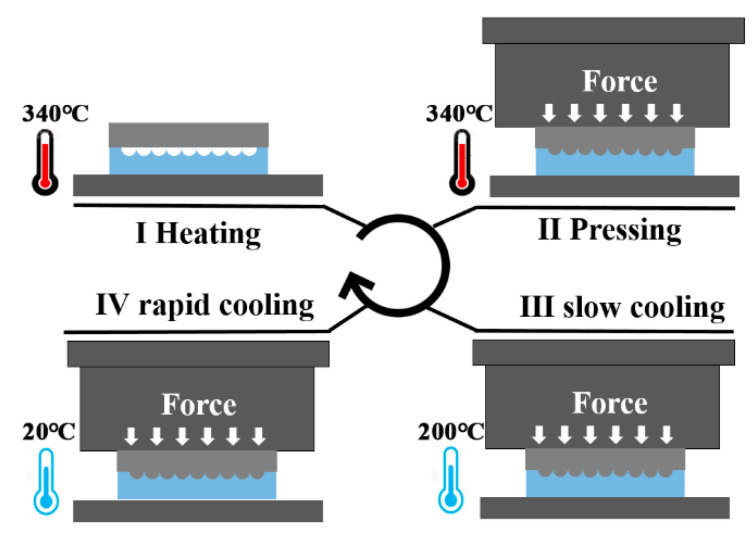
Schematic diagrams of the molding process: I heating, II pressing, III slow cooling, IV rapid cooling.

With precision glass molding, a piece of ChG was used to replicate the convex IR MLA. The pre-dried ChG was sandwiched between the mold and a glass substrate, then the assembled module was installed in the high-temperature furnace. The molding process was divided into four stages: the heating stage, the pressing stage, the slow cooling stage and the rapid cooling stage, as shown in Figure 2 [47]. At each stage, the precision glass molding processes was optimized as follows: (1) the mold and ChG were heated to the specific temperature of 340 °C at a temperature increment of 8 °C/min; (2) when the specific temperature was reached, a pressure of 500 kPa was vertically applied onto the mold and kept for 10 min; (3) the pressed MLA was cooled to release the pressure induced inside the ChG by a slow cooling stage to 200 °C with temperature decrement of 2 °C/min; and (4) the MLA was rapidly cooled to room temperature with a temperature decrement of 10 °C/min, shown in Figure 1d. The replicated ChG MLA was demolded at room temperature (Figure 1f). Finally, convex MLAs with a nanometer scale surface roughness (49 nm) were successfully fabricated on the surface of ChG, shown in Figure 1e.

## 3. Results

The molding process parameters were determined based on a previous experiment and our parameter optimization experiments that examined the effect of temperature on the moldability of ChG, as shown in Table 2. The time of the glass molding process was 138 min. The temperature increment should not be too high so as to prevent the molded MLAs from breaking. When the temperature of the pressing stage was higher than the softening point, the molding loads of the pressing and slow cooling stages remained at 500 kPa. Under these imprinting parameters, MLAs did not break, and the glass did not adhere to the mold surface.

**Figure 3:**
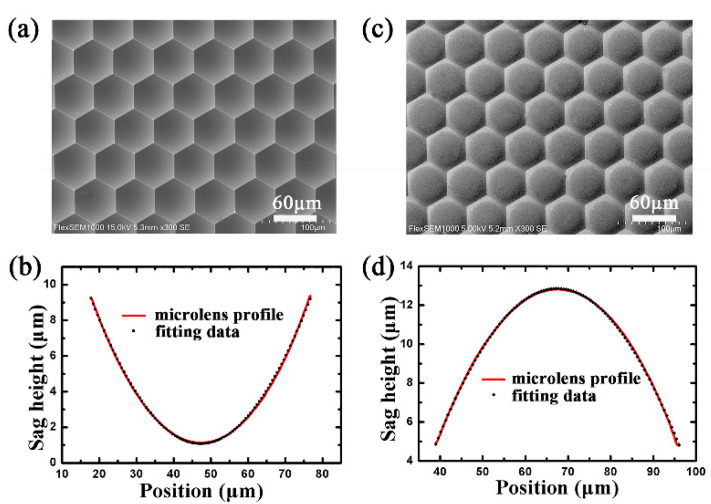
(**a**) SEM image of master mold. (**b**) Cross-sectional profile of a concave microlens mold. (**c**) SEM image of ChG convex microlenses. (**d**) Cross-sectional profile of ChG convex microlenses.

To gain a convex ChG MLA, replication based on precision glass molding was implemented. During this process, thousands of ChG convex MLAs could be replicated from the master mold without forming visible defects. Figure 3a,b shows the scanning electron microscopy (SEM) images of the master mold MLAs and the molded MLAs, in which a close-packed microstructure without gaps can be observed. Therefore, the fill factor of the MLAs reached 99%, which was critical to gather more light and improve the sensitivity for the application of the uncooled IR thermal imaging and sensor systems. To study the surface profile of the convex microlens, we used conic curves to fit the cross-sectional profile. Figure 3c,d shows the cross-sectional profile of the microlens (line) and the ideal parabola (dots). It is reasonable to determine that the cross-sectional profile is a parabola. The focal length of the convex microlens can be calculated as (Equation (1)) [48,49]:

(1) $$f=\frac{h^{2}+r^{2}}{2h\left(n-1\right)}$$

where *h* is the sag height of the microlens, *n* is the refractive index of ChG, and *r* is the aperture radius of the microlens. Given *h* = 8.008 μm, *r* = 28.75 μm, and *n* = 2.58, we have *f* = 35.2 μm.

**Figure 4:**
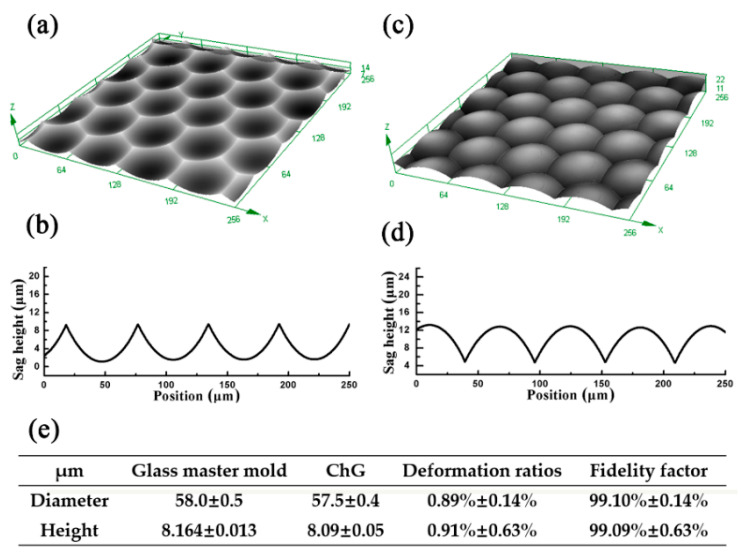
(**a**,**b**) The 3D morphology and cross-section of the concave MLAs; (**c**,**d**) The 3D morphology and cross-section of the convex MLAs; (**e**) The fidelity factor of convex MLAs.

To investigate the 3D morphology, the microlens of the master mold and the molded MLAs were measured by a laser scanning confocal microscope (LSCM) (OLS4000, Olympus Corporation, Tokyo, Japan) The results are shown in Figure 4a,c. The optically smooth, large-area and close-packed convex MLAs could be fabricated by the precision glass molding process. Moreover, the 2D profiles of the microlenses were quantified by LSCM to investigate the fidelity ratio, as shown in Figure 4b,d. In order to confirm the transcription characteristics of the ChGs surface, different regions of the concave MLA master mold and the convex MLA were measured, and the data were statistically processed and analyzed. Consequently, the average aperture diameters and sage heights of the mold microlenses were 58 and 8.173 μm, and the average aperture diameters and sage heights of the molded microlenses were 57.5 and 8.008 μm. The deformation ratios were ≈ 2% (Figure 4e), demonstrating that this simple method could realize convex MLAs on the surface of ChG with relatively high fidelity.

**Figure 5:**
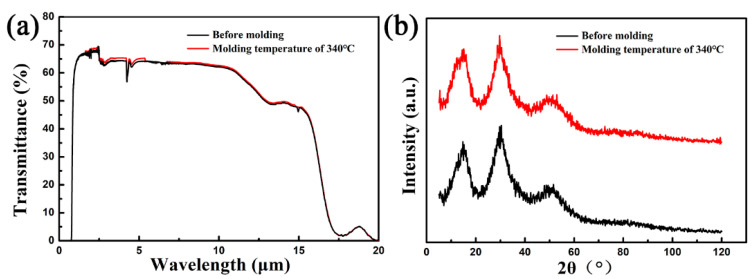
(**a**) Transmittance of the ChG before and after molding. (**b**) XRD patterns of the ChG before and after molding.

In order to verify the compositional and structural stability of ChG under the given molding conditions, the IR transmittance (Nicolet iS10, Thermo Fisher Corporation, Waltham, MA, USA) and x-ray diffraction (XRD) (D8 Advance A25, Bruker Corporation, Karlsruhe, Germany) image of the ChG MLAs were measured. Figure 5a shows the transmittance of the ChG before molding and the MLAs molded at 340 °C in the spectral range of 2 to 20 μm. The transmittance of the molded MLAs was similar to that of the ChG before molding. The XRD analysis of the ChG before and after molding is shown in Figure 5b. No additional peaks were found in the ChG at 340 °C. The IR transmittance and the XRD analysis showed that the composition and structure of ChG were stable under the corresponding molding condition.

**Figure 6:**
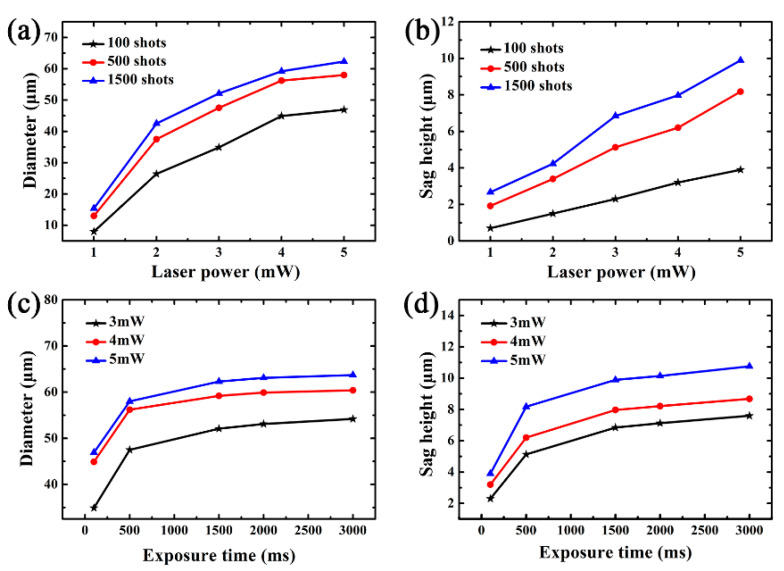
(**a**) The power dependency of the diameter of the concave structures. (**b**) The power dependency of the sag height of the concave structures. (**c**) The relationship between the diameter and laser exposure time. (**d**) The relationship between the sag height and the laser exposure time.

This work reveals a flexible method for fabricating microlens arrays of various sizes. The diameters and sag heights of the mold MLA can be easily tuned and controlled by adjusting the laser irradiation conditions of the femtosecond laser-assist chemical etching process. We fabricated microlenses at different laser powers and exposure times, shown in Figure 6. In Figure 6a,b, the diameters and sag heights produced by 1, 2, 3, 4 and 5 mW laser powers are shown from left to right. This indicates that the diameter and sag height of the concave MLA were closely related to the laser power. The relationships between laser exposure time and microlens sizes were also studied by a similar method. The results shown in Figure 6c,d demonstrate that both diameter and sag height of the MLA were weakly dependent on laser exposure time. From the above, FLACE is a simple, highly efficient and maskless technique to create concave MLAs. By means of this method, large-area concave MLAs were fabricated for one hour, and the fabricated MLAs had excellent surface quality and uniformity. In contrast to the classic methods, FLACE can flexibly control the size of the MLAS by adjusting the laser parameters. Although this method can only process concave MLAs, it meets the needs of the experimental requirements.

**Figure 7:**
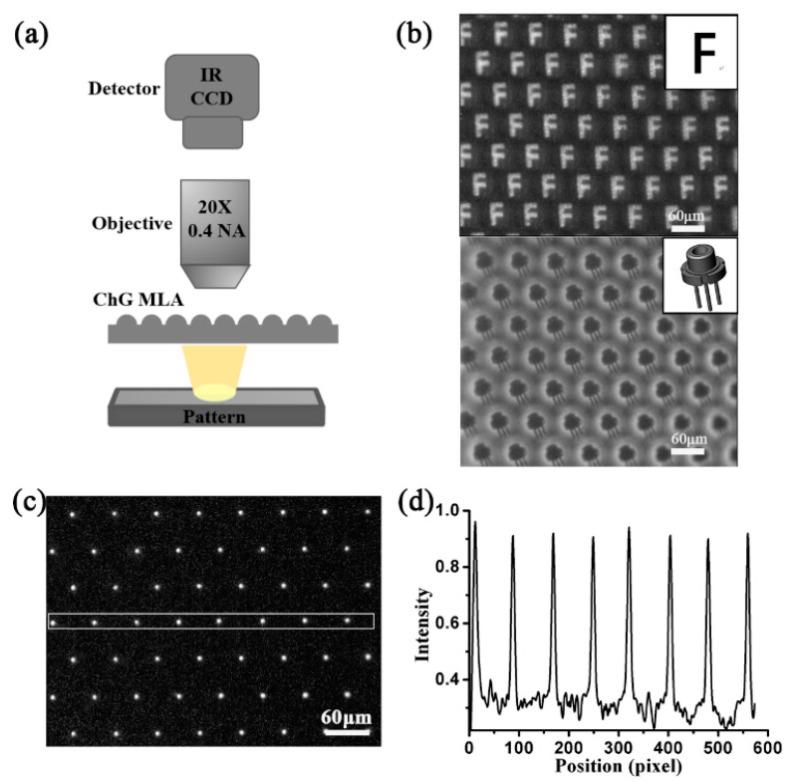
(**a**) The optical characterization IR system for ChG MLAs. (**b**) Clear IR images obtained from the ChG MLAs. (**c**) The focal point is observed with an IR charge coupled device (CCD), and the focal length is measured to be about 34 μm. (**d**) The normalized intensity distribution obtained in the selected rectangular area.

To evaluate their ability to act as IR microlenses, we set up an IR optical system using ChG MLAs. Figure 7a shows the schematic illustration of the system, which mainly consists of an IR optical microscope, the ChG MLAs and a wide spectral light source. When we placed patterned objects (a black sheet with a transparent pattern on it) between the light source and the ChG MLAs, the images shown in Figure 7b were clearly observed. Because of the low surface roughness and low aberrations of the ChG MLAs, the generated IR images were sharp and exhibited high contrast. As shown in Figure 7b (the lower image), the pins of the triode can be well-recognized, indicating the good resolving ability of the ChG MLAs. In addition, for demonstrating the unique focusing characteristic more clearly, a focus imaged was obtained (Figure 7c). Each microlens can form a clear and circular focusing spot. The average focal length *f* of the microlens was measured to be 34 ± 3 μm, which agreed with the calculated value. The cross-sectional intensity distribution of the focusing spots in the selected rectangular area was calculated, as shown in Figure 7d. The sharp intensity distribution of the focal points indicated that each microlens was focused well.

## 4. Conclusions

We have successfully fabricated high-quality, infrared, convex MLAs using precision glass molding technology, and it has the merits of being micro-sized, lightweight, having high integration, high IR transmittance and excellent IR imaging performance. The conclusions could be summarized as follows: (1) A high-quality microlens array mold with high fill-factor, controllable size and complex shape was fabricated on a hard and high-temperature-resistant BK7 glass via FLACE. (2) By precisely controlling rates of heating and cooling in the four stages, we found no MLA breakage or adhesion of the ChG to the mold surface. (3) The IR transmittance and x-ray diffraction analysis of the ChG MLAs showed that the structure and composition of the ChG were stable at molding temperatures of 340 °C. (4) This was a flexible method to fabricate CHG MLAs of various sizes by changing the laser irradiation conditions. The CHG MLAs have relatively good topography, surface quality, and high transparency at wavelengths of 2.5–20 μm. All the evidence proves that precision ChG molding is a viable technology for IR imaging and IR sensing applications.

## Figures and Tables

**Table 1:** Thermal and mechanical properties of chalcogenide glass (NBU-IR1).

Physical Properties	Value
Thermal conductivity (Wm^−1^K^−1^)	0.23
Thermal expansion coefficient (K^−1^)	1.41 × 10^−5^
Transition temperature (T_g_) (K)	558.15
Softening point (T_s_) (K)	578.15
Refractive index at 10 μm	2.58609 (298.15 K), 2.58928 (301.15 K)
Thermo-optic coefficient at 10 μm (K^−1^)	5.8 × 10^−5^
Young’s modulus (GPa)	19.11

**Table 2:** Nano-imprinting conditions and process parameters used in this study.

Stage	Heating	Pressing	Slow Cooling	Rapid Cooling
Temperature (°C)	340	340	200	20
Pressing force (kPa)	0	500	500	500
Process time (min)	40	10	70	18
